# Piezoelectric micromanipulator dataset for hysteresis identification

**DOI:** 10.1016/j.dib.2020.105175

**Published:** 2020-01-25

**Authors:** Helon Vicente Hultmann Ayala, Micky Rakotondrabe, Leandro dos Santos Coelho

**Affiliations:** a Department of Mechanical Engineering, Pontifical Catholic University of Rio de Janeiro, Brazil; b Laboratoire Génie de Production, National School of Engineering in Tarbes / INPT, France; c Industrial and Systems Graduate Program, Pontifical Catholic University of Paraná, Brazil

**Keywords:** Micromanipulators, System identification, Piezoelectric actuator, Mechatronics

## Abstract

This data in brief presents displacement measurements acquired from a piezoelectric cantilevered actuator when subjected to harmonic excitations. The micro displacements are measured with optical sensors. The dataset has been used recently for the purpose of nonlinear black-box modelling, where the hysteretic behaviour of such devices has been modelled [1,2]. We hope to enable reproducibility by sharing the data used in [1,2], which are previous works by the authors, allowing the comparison of new methods on a common basis. Additionally, researchers interested in piezoelectric actuators for high precision tasks may also benefit on working with the present dataset.

**Table:** Specifications Table

Subject	Control and Systems Engineering
Specific subject area	System identification and hysteresis modelling
Type of data	Deflection and displacement measurements
How data were acquired	Optical sensors
Data format	Raw
Parameters for data collection	The piezoelectric actuator was excited using a sine driving voltage. The amplitudes and frequencies tested were 5V and 150 V and 300Hz and 1 Hz, respectively.
Description of data collection	The entire test bench constructed to collect data is composed of (a) the piezoelectric actuator, (b) an optical sensor (LK2420 from Keyence company) which is employed to measure the deflection (displacement) of the above actuator and has been tuned to have 10nm resolution and in excess of 5kHz bandwidth, (c) a computer which is used to generate the sine driving voltage and to acquire the measurement from the optical sensor, (d) a dSPACE (type DS1104) acquisition board that serves as digital-to-analogic and as analogic-to-digital converters between the computer and the rest of the physical setup, with sampling period set as 50 μs, and (e) a high voltage amplifier that multiplies by 20 the driving voltage from the computer before sending it to the actuator.
Data source location	Institution: ENIT/Toulouse University, University of Toulouse City/Town/Region: Tarbes Country: France
Data accessibility	With the article
Related research article	Helon Vicente Hultmann Ayala, Didace Habineza, Micky Rakotondrabe, Leandro dos Santos Coelho, Nonlinear Black-box System Identification through Coevolutionary Algorithms and Radial Basis Function Artificial Neural Networks, Applied Soft Computing, vol. 87, 105990, 2020.

**Table:** 

**Value of the Data** • The dataset provided is important for nonlinear modelling of hysteretic systems • Researchers in the system identification community at large may benefit for testing nonlinear modelling techniques. Researchers and engineers working with piezoelectric actuators for high precision positioning applications may also be interested with and benefit from the data. • The present dataset enable comparison among methods for modelling a phenomenon that is frequently found in positioning applications, but not only.

## 1 Data description

The dataset is composed of two input/output data pairs. The system is excited with a sine voltage input of (a) 150 V and 1 Hz; and (b) 5 V and 300 Hz. The dataset (b) has been employed for identification in Refs. [1,2]. The goal of measuring both datasets is to evaluate the modelling activity when working under different frequencies and amplitudes. Dataset (a) has not been explored thus far in any publications.

The datasets (a) and (b) are provided in two comma separated values (CSV) files. In these files, which can be visualized in any text editor, each line refers to a sampling time instant. Both CSV files are zipped in a single file, which is provided as a supplement to this article. In this zip file, there is also a MATLAB code to plot the data. It is possible to find below a detailed description of both datasets, according to their filename:

(a) **h50us.csv**: this dataset contains 200,001 measurements sampled in time every 50 microseconds. The second, third, and fourth columns are respectively the vector with time, output displacement, and input voltage. The measurements are drifted, so a pre-processing is needed (in the code provided this is already arranged).
(b) **hysteresis_v_150_1hz.csv**: the second dataset has the same file structure with respect to the columns as (a) but measured every 20 milliseconds with 50,001 samples. The sinusoidal signal starts at approximately 3.25 seconds, so the first samples of the dataset should be discarded (in the code provided this is already arranged).

**Fig. 1:**
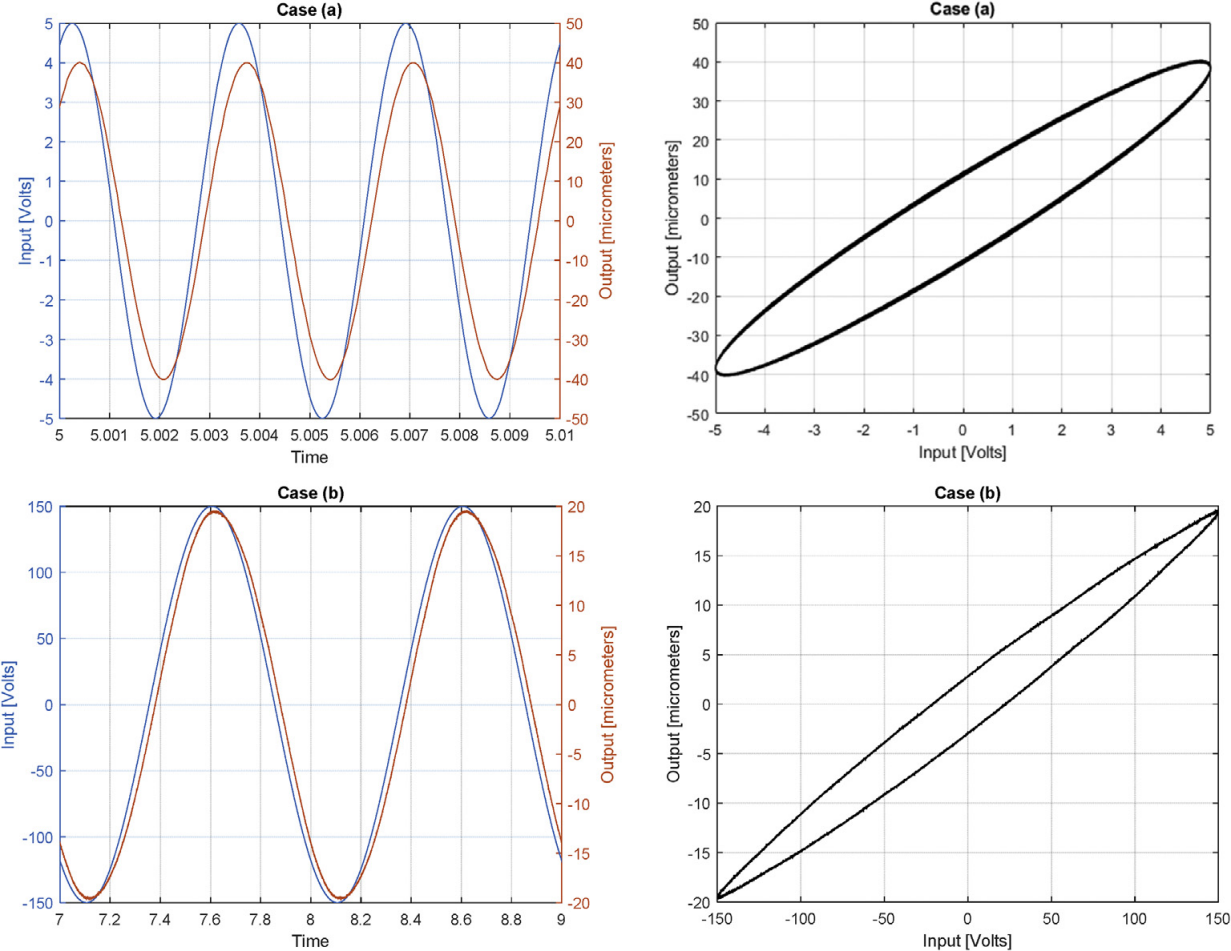
Input and output data for case (a) – top - and (b) - bottom. On the left we see the time histories for both input and output and on the right the semi-static curves (input vs. output) are given.

Datasets (a) and (b) characteristics are summarized in Table 1 and depicted graphically in Fig. 1. They are sampled at different rates as the dynamics due to the excitation is faster in case (a). It is possible to see that the voltage amplitudes and frequencies are different for each file, as Table 1 shows. Nonetheless, the amplitudes for the deflections are in the same order of magnitude, as the input/output gain for each frequency is different for the system. For the sinusoidal-like type of input, it is possible to plot the graph with input versus output through time, where the hysteretic behaviour can be clearly observed.

**Table 1:** Dataset characteristics for each file provided.

Dataset	Input Amplitude [V]	Input Frequency [Hz]	Sampling Frequency [kHz]	Time [s]
(a)	5	300	20	10
(b)	150	1	5	10

## 2 Experimental design, materials, and methods

**Fig. 2:**
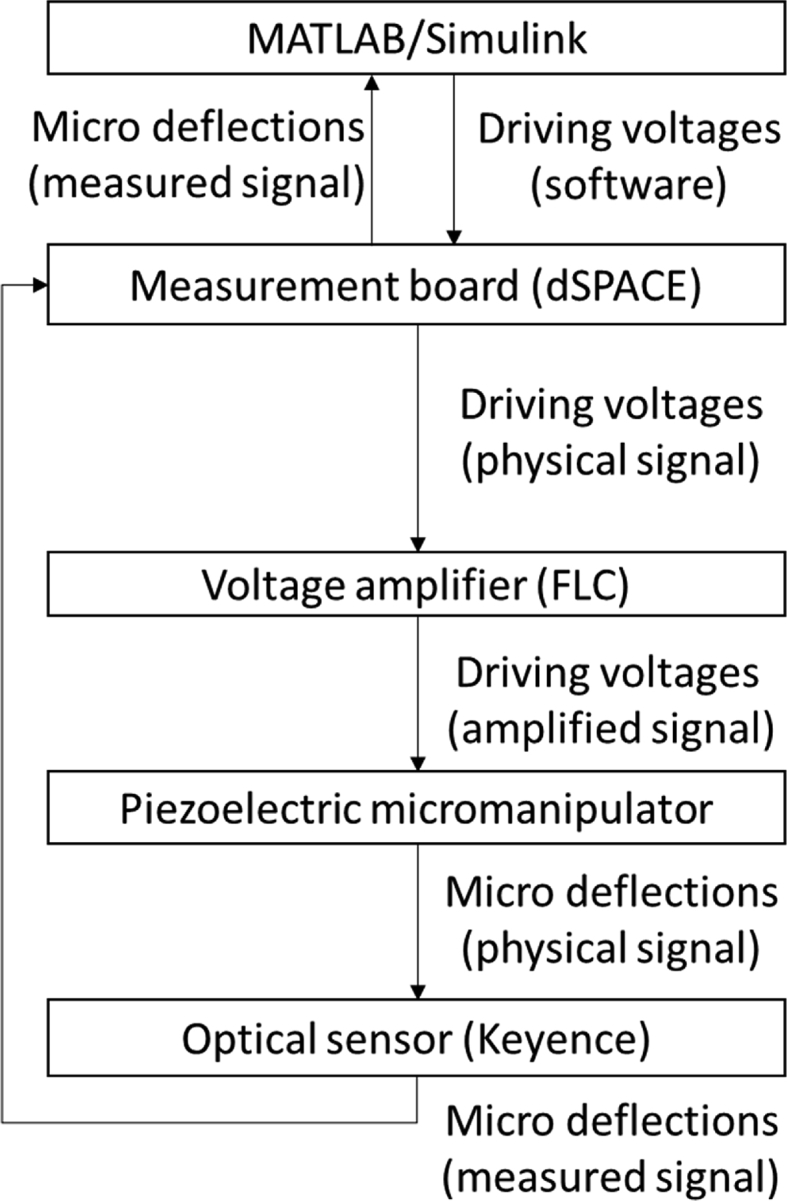
Schematic of the measurement setup and the data flow among its components.

**Fig. 3:**
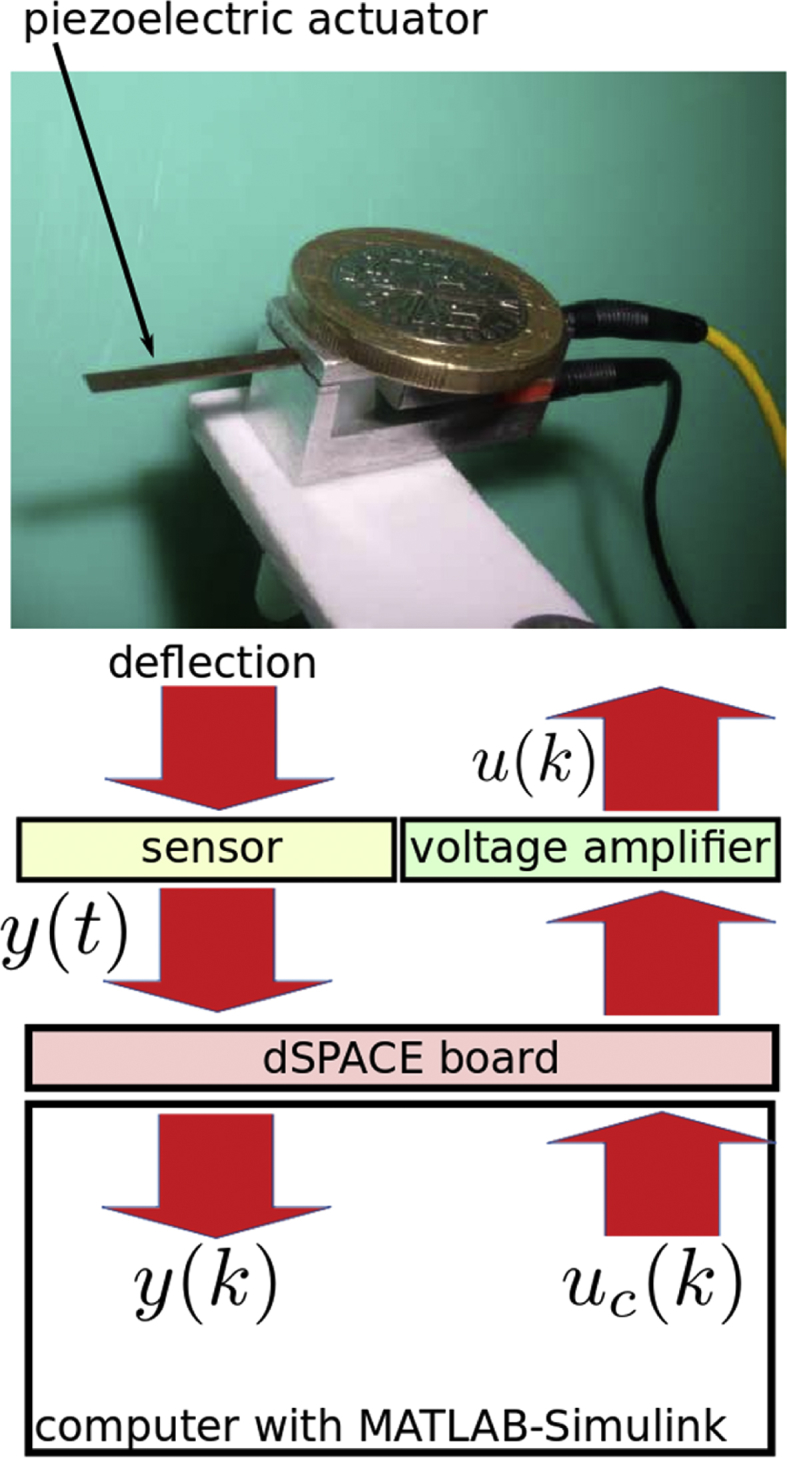
Description of the piezoelectric micromanipulator and its measurement interfaces.

A schematic of the experimental benchmark setup is described in Fig. 2. The components used in this setup are described in detail in Table 2. The piezoelectric micromanipulator is manufactured with 15 × 2 x 0.3 (length, width, and thickness, in millimetres), where the piezoelectric and passive layers have, respectively, 0.2 and 0.1 mm. For a real picture of the setup with the measurement device, please refer to Fig. 3.

**Table 2:** Hardware used for instrumentation of the test bench and respective purpose description.

Hardware	Manufacturer	Purpose
DS1104	dSPACE	- converts the sine driving voltage generated from MATLAB-Simulink in the computer into analogic voltage outside the computer, - converts the measured displacement from the sensor into numeric measurement inside MATLAB-Simulink.
LK2420	Keyence	Optical sensor that measures the deflection (displacement) of the piezoelectric actuator.
A400DI	FLC	Amplifies the voltage from the acquisition board and computer before driving the piezoelectric actuator.
Computer (with MATLAB-Simulink)	Any	MATLAB-Simulink has been used to program the voltage to be amplified and sent to the actuator and used to save or display the measurement from the sensor.

The dataset is provided with a MATLAB code (read_plot_data.m) that reads the data into memory and plots the graphs given in Fig. 1.
